# Association between HBs Ag quantification and the risk of hepatocellular carcinoma in patients treated with tenofovir disoproxil fumarate or entecavir

**DOI:** 10.1097/MD.0000000000027417

**Published:** 2021-10-01

**Authors:** Jung Hyun Lim, Jung Hwan Yu, Young Ju Suh, Jin-Woo Lee, Young-Joo Jin

**Affiliations:** a Department of Internal Medicine and Division of GI and Liver Diseases, Inha University Hospital, Inha University School of Medicine, Incheon, South Korea; b Department of Statistics, Inha University Hospital, Incheon, South Korea.

**Keywords:** chronic hepatitis B, entecavir, HBs Ag quantification, hepatocellular carcinoma, tenofovir disoproxil fumarate

## Abstract

This study evaluated the clinical implications of hepatitis B surface antigen quantification (qHBs Ag) in chronic hepatitis B (CHB) patients treated with entecavir (ETV) or tenofovir disoproxil fumarate (TDF) and identified the association between qHBs Ag and the risk of hepatocellular carcinoma (HCC) in these patients.

Between January 2007 and December 2018, the qHBs Ag and clinical data of 183 CHB patients who initially received ETV (n = 45, 24.6%) or TDF (n = 138, 75.4%) were analyzed.

The mean follow-up period of the 183 CHB patients was 45.3 months, of which 59 (32.2%) patients showed a reduction in qHBs Ag by >50% after 1 year of antiviral treatment (ETV or TDF). The HCC development (*P* = .179) or qHBs Ag reduction (*P* = .524) were similar in the ETV and TDF groups. Patients with a ≥50% decrease in qHBs Ag had a significantly lower incidence of HCC or decompensated cirrhosis complications (*P* = .005). Multivariate analysis showed that a >50% reduction of qHBs Ag (hazard ratio 0.085, *P* = .018) and the presence of cirrhosis (hazard ratio 3.32, *P* = .016) were independent factors predicting the development of HCC.

Patients whose qHBs Ag value decreased >50% at 1 year after antiviral treatment for CHB showed a significant decrease in HCC or decompensated cirrhosis events. A reduction in qHBs Ag could be used as a predictive factor of HCC development or critical complications in CHB patients treated with TDF or ETV.

## 1 Introduction

Hepatitis B virus (HBV) is a leading cause of cirrhosis and hepatocellular carcinoma (HCC) and is known to affect more than 2.5 million people worldwide.^[1]^ Since HCC is closely associated with chronic hepatitis B (CHB) infection,^[2,3]^ oral antiviral therapy for HBV can improve the prognoses of CHB patients by preventing the development of HCC and decompensated complications of cirrhosis.^[4]^ In particular, drugs with a high barrier to resistance, such as entecavir (ETV) or tenofovir (TDF),^[5,6]^ which are recommended as a first treatment drugs for CHB,^[7–10]^ have a long-term antiviral effects in more than 95% of CHB patients, and have the effect of restoring liver function.^[11–13]^ On the other hand, despite the use of highly potent antiviral drugs, the risk of HCC remain in patients with CHB.^[14–16]^ Therefore, assessing the risk of HCC in these patients and finding the factors associated with HCC is an important issue.

HBs Ag quantification (qHBs Ag) has been studied to date primarily as a predictor of HBe Ag seroconversion or HBs Ag clearance after pegylated interferon-alpha (Peg-IFN-α) or nucleos(t)ides analogue therapy.^[17–19]^ Recently, however, qHBs Ag has become increasingly recognized as a marker for predicting HCC and the development of cirrhosis in CHB patients.^[20,21]^ A recent meta-analysis involving 6028 patients with CHB reported a greater risk of HCC in patients with high serum HBsAg levels (≥1000 IU/mL) than that among patients with low HBs Ag levels.^[22]^ Similarly, a systemic review showed that the cumulative incidence of HCC over a 14-year follow-up increased when HBs Ag was higher in a dose-response manner.^[20]^ However, studies on the risk of qHBs Ag and HCC in CHB patients treated with highly potent antiviral drugs, such as ETV and TDF, which are used widely, are still lacking. Moreover, little is known regarding the differences between these 2 antiviral agents in qHBs Ag reduction.

Therefore, in this study, we intended to assess the predictive power of qHBs Ag for HCC development and critical event risk in CHB patients initially treated with ETV or TDF, and to identify the clinical implication of HBs Ag in these patients. In addition, we tried to find out the difference in the reduction of HBs Ag between the 2 drugs, ETV and TDF.

## 2 Patients and methods

### 2.1 Study subjects

A study population of 899 CHB patients registered between January 2007 and December 2018 received ETV or TDF as an initial treatment for CHB infection. All of them had been HBs Ag-positive for longer than six months and had undergone antiviral therapy for a CHB infection according to the guidelines published by the American Association for the Study of Liver Diseases (AASLD).^[7]^ The HBs Ag quantitative values of all enrolled patients were measured immediately before ETV or TDF administration, and then every 6 months at a regular follow-up for >5 years at Inha University Hospital. The method of measuring the HBs Ag quantitative value in all patients was the Architect HBs Ag QT (Abbott Diagnostic, Wiesbaden, Germany). ETV and TDF have been available at the authors’ hospital since 2007 and 2012, respectively, after receiving certification for compliance with the Korean insurance coverage standards. Therefore, patients who took ETV and TDF could receive a prescription from 2007 and 2012, respectively.

**Figure 1:**
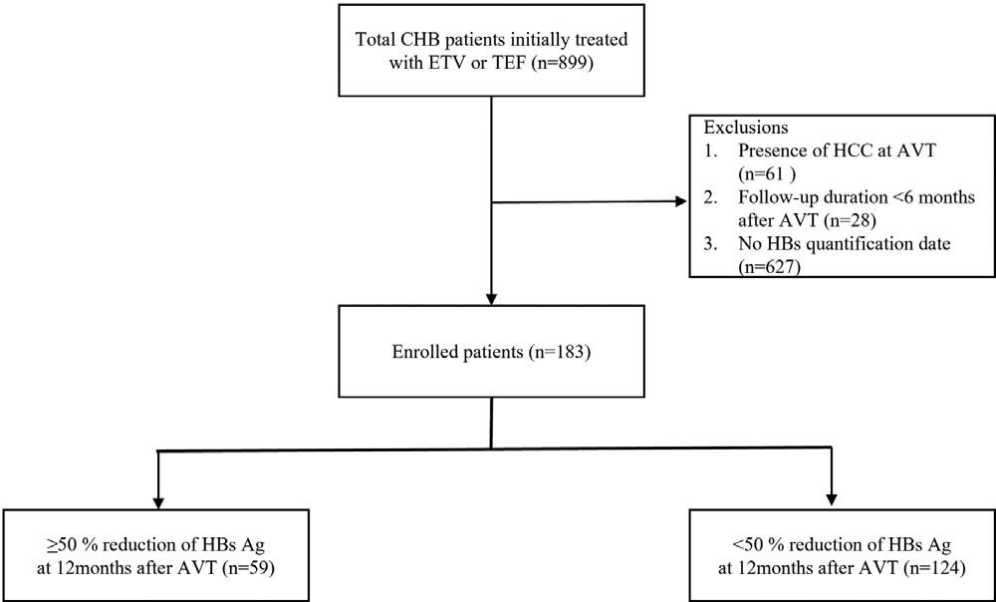
Study subjects. Of the 899 chronic hepatitis B (CHB) patients treated at our institution from January, 2007 to December, 2018, 183 patients treated with entecavir (ETV) (n = 45, 24.6%) or tenofovir disoproxil fumarate (TDF) (n = 138, 75.4%) were enrolled in this study.

Patients with another chronic liver disease, such as hepatitis C virus infection, alcoholic liver disease, primary biliary cirrhosis, Wilson disease, autoimmune hepatitis, or those aged ≤18 years were excluded. In addition, patients with a current HCC or another malignancy at the start of antiviral therapy (n = 61) or with a follow-up duration <6 months after antiviral therapy (n = 28) were excluded. All patients whose HBs Ag quantitative values were missing from the beginning (n = 627) were also excluded (Fig. 1). Accordingly, 183 CHB patients treated with ETV (n = 45, 24.5%) or TDF (n = 138, 75.4%) were enrolled in this study, and their electronic medical records were analyzed retrospectively. The study was approved by the Institutional Review Board of Inha University Hospital, Incheon, South Korea (Approval number: INHAUH 2020-11-014).

### 2.2 Recruitment of clinical data

The demographic data of the patients, liver function tests, and presence of liver cirrhosis, serum alpha-fetoprotein (AFP) level, Child-Turcotte-Pugh classification, hepatitis B envelope antigen (HBe Ag) and its seroconversion, and HBV DNA (Copies/mL) levels were collected from the electronic medical records and clinical history. The quantitative value of HBs Ag (IU/mL) was measured using Abbott quantification device at Inha University Hospital. The HBV DNA quantitative level was measured using a hybridization assay (a detection limit of 2000 copies/mL) at the start of antiviral therapy. Liver cirrhosis was diagnosed from the clinical evidence of portal hypertension (esophageal varices, ascites, encephalopathy, splenomegaly, or low platelet count,) or by liver ultrasonography.^[23,24]^

### 2.3 Surveillance of HCC

To assess HCC development, the patients were routinely tested for the serum AFP and liver US or liver computed tomography (CT) every 6 months to 1 year. The follow-ups started from the start of antiviral therapy and continued until the date of HCC diagnosis or the last follow-up, or April 30, 2020. HCC was diagnosed based on the guidelines published by the AASLD.^[2]^ If HCC developed after starting antiviral therapy, the tumor size or number, tumor type (nodular or infiltrative), presence of portal vein thrombosis or extrahepatic metastasis, serum AFP level at the time of the HCC diagnosis, and BCLC (Barcelona Clinic Liver Cancer) stage were obtained. Chest CT and a bone scan were performed to detect any extrahepatic metastasis.

### 2.4 Statistical analyses

The baseline clinical characteristics are described as the median (range) or frequency. Differences between the categorical or continuous variables were analyzed using a *χ*^2^ test, Fisher exact test, or the Student *t* test. The independent risk factors of HCC were identified by multivariate analysis using a logistic regression model. The odds ratios (OR) with 95% confidence intervals (CI) were calculated using this model. The cumulative HCC development rates were estimated by Kaplan–Meier analysis, and the log-rank test was used to compare the data among the groups. Two-tailed *P* values of <.05 were considered significant, and statistical analysis was performed using SPSS v19.0 (SPSS Inc, Chicago, IL).

## 3 Results

### 3.1 Baseline characteristics

Table 1 lists the baseline characteristics of the 183 patients. The median patient age was 49.4 years (range, 18–79 years), and 97 (53%) were male. LC was present in 79 (43.2%) patients, and most (97.4%) of these patients were in a compensated state with Child-Turcotte-Pugh class A or B. HBe Ag was positive in 95 (51.9%) patients. The median follow-up duration was 45.4 months (range, 3.1–96.6 months). The study subjects were divided into 2 groups: one showed a >50% reduction in quantitative HBs Ag (qHBs Ag) levels after 1 year of antiviral treatment (reduction group), and the other group showing a reduction of less than 50% or an increase (nonreduction group). Of the 183 patients, 124 (67.8%) patients were in the nonreduction group, and 59 (32.2%) patients were in the reduction group. At the start time of antiviral therapy, there were no significant differences in both groups. Only the average follow-up duration was 6 months longer in the reduction group (49.7 months) than the nonreduction group (43.4 months) (*P* = .046).

**Table 1:** Baseline clinical characteristics of study subjects.

Variables	Total (n = 183)	Non 50% reduction group (n = 124, 67.8%)	50% Reduction group (n = 59, 32.2%)	*P* ^∗^
Age, y^†^	49.4 (18–79)	49.0 (18–74)	50.4 (20–79)	.473
Sex (male), n (%)	97 (53.0)	69 (55.7)	28 (47.5)	.343
ALT, IU/L^†^	162.0 (13–1871)	146.6 (13–1871)	194.5 (14–1060)	.206
Albumin, mg/dL^†^	4.01 (1.9–5.2)	4.0 (1.9–5.0)	4.03 (2.4–5.2)	.758
T-bil, mg/dL^†^	1.47 (0.1–38.2)	1.16 (0.1–18.8)	2.13 (0.3–38.2)	.081
PT, INR^†^	1.12 (0.87–2.07)	1.11 (0.87–2.07)	1.14 (0.9–2.06)	.314
AFP, ng/mL^†^	42.9 (0.8–1391)	37.8 (0.8–1153)	53.7 (0.9–1391)	.543
LC, present, n (%)	79 (43.2)	54 (43.5)	25 (42.4)	1.000
CTP class, A/B/C, n (%)	154/27/2 (84.2/14.8/1.0)	107/16/1 (86.3/12.9/0.8)	47/11/1 (79.7/18.6/1.7)	.664
HBeAg, positive, n (%)	95 (51.9)	64 (51.6)	31 (52.5)	1.000
HBeAg loss at 12 mo, yes, n (%)	15 (8.2)	9 (7.3)	6 (10.2)	.555
HBV-DNA, copies/mL^†^	3.16 × 10^8^ (57–3.41 × 10^9^)	1.60 × 10^8^ (57–3.41 × 10^9^)	6.43 × 10^8^ (95–3.41 × 10^9^)	.000
HBV-DNA negative at 12 mo, yes, n (%)	146 (79.8)	102 (82.2)	44 (74.6)	.242
FU duration, mo,^†^	45.4 (3.1–96.6)	43.3 (3.1–84.3)	49.7 (4.1–96.6)	.046

AFP = alpha-fetoprotein, ALT = alanine aminotransferase, CTP = child-turcotte-pugh, DNA = deoxyribonucleic acid, ETV = entecavir, FU = follow-up, HBeAg = hepatitis B envelope antigen, HBV = hepatitis B virus, INR = international ratio, LC = liver cirrhosis, PT = prothrombin time, T-bil = total bilirubin, TDF = tenofovir disoproxil fumarate.

∗ *P* values were calculated using the *t* test or *χ*^2^ test between a non-50% reduction and a 50% reduction group.

† Median (range).

### 3.2 Cumulative critical event development in CHB patients treated with ETV or TDF

A critical event that can occur due to a CHB infection was defined in the present study as the development of decompensation (ascites, variceal bleeding, jaundice, spontaneous bacterial peritonitis, and hepatic encephalopathy), the occurrence of HCC, and death. Over the median follow-up period of 45.4 months, critical events developed in 22 (12.0%) of the 183 study subjects, regardless of antiviral agents used (Table 2). Of them, 15 patients (68%) had cirrhosis at a relatively larger rate than the no critical event group (*P* = .020), as expected. Among the hematological tests, albumin, the total bilirubin, PT (INR), and AFP did not significantly differ between the 2 groups. Only ALT showed higher patterns in the noncritical event group (*P* = .019).

**Table 2:** Characteristics of CHB patients with critical event.

Variables	Total (n = 183)	Event group (n = 22, 12.0%)	No event group (n = 161, 88.0%)	*P* ^∗^
Age, years^†^	49.4 (15–79)	47.7 (24–70)	49.7 (15–79)	.486
Sex (male), n (%)	97 (53.0)	15 (68.2)	82 (50.9)	.097
ALT, IU/L^†^	162.0 (13–1871)	50.8 (13–206)	177.2 (14–1871)	.019
Albumin, mg/dL^†^	4.0 (1.9–5.2)	3.9 (2.3–4.6)	4.0 (1.9–5.2)	.178
T-bil, mg/dL^†^	1.5 (0.1–38.2)	1.1 (0.3–2.9)	1.5 (0.1–38.2)	.552
PT, INR^†^	1.12 (0.87–2.07)	1.17 (0.9–1.65)	1.11 (0.87–2.07)	.189
AFP, ng/mL^†^	42.9 (0.8–1391)	16.6 (1.4–202.5)	46.5 (0.8–1391)	.425
LC, present, n (%)	79 (43.2)	15 (68.2)	64 (39.8)	.020
CTP class, A/B/C, n (%)	154/27/2 (84.2/14.8/1.1)	15/7/0 (68.2/31.8/0)	139/20/2 (86.3/12.4/1.2)	.051
HBeAg, positive, n (%)	95 (51.9)	10 (45.5)	85 (52.8)	.650
HBeAg loss at 12 mo, yes, n (%)	15 (8.2)	1 (4.5)	14 (8.7)	1.000
HBV-DNA, copies/mL^†^	3.16 × 10^8^ (57–3.41 × 10^9^)	1.6 × 10^7^ (57–1.5 × 10^8^)	3.57 × 10^8^ (88–3.41 × 10^9^)	.061
HBV-DNA negative at 12 mo, yes, n (%)	146 (79.8)	18 (81.8)	128 (79.5)	1.000
>50% Deceased HBsAg at 12 mo, yes, n (%)	59 (32.2)	1 (4.5)	58 (36.0)	.003

AFP = alpha-fetoprotein, ALT = alanine aminotransferase, CHB = chronic hepatitis B, CTP = Child-Turcotte-Pugh classification, HBs Ag = hepatitis B surface antigen, INR = international normalized ratio, LC = liver cirrhosis, PT = prothrombin time.

∗ *P* values were calculated using the *t* test or *χ*^2^ test between the event and no event group.

† Median (range).

**Figure 2:**
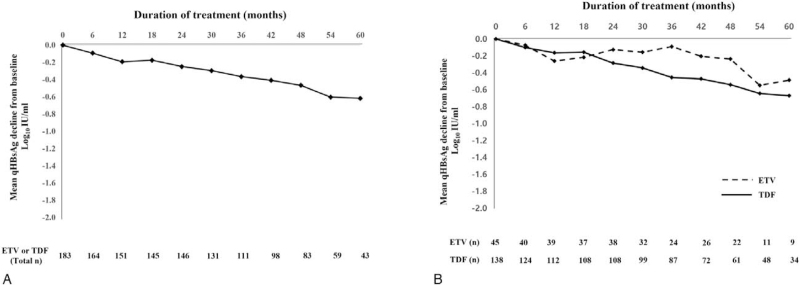
Mean hepatitis B surface antigen quantification decline from baseline in all study subjects During a median follow-up of 45.4 months, the entecavir (ETV) and tenofivir disoproxil fumarate (TDF) patients groups showed similar hepatitis B surface antigen quantification (qHBs Ag) decline during 1 year after antiviral therapy (*P* = .205). Mean qHBs Ag decline from baseline without dividing ETV and TDF patients groups (A) and with dividing ETV and TDF patients groups (B).

Graphing the difference in the qHBs Ag log values for all patients over 5 years confirmed an overall decrease. Figure 2A shows the mean qHBs Ag decline from baseline for all patients and Figure 2B shows the mean decline divided between ETV-treated patients and TDF-treated patients (Fig. 2). The data in Figure 2 panel B seems to show a more consistent decrease over time in the group of patients taking TDF. However, the number of patients who decreased by <50% or increased in the ETV group was not significantly greater than that of the TDF group (*P* = .205). There was no statistically significant difference, but the number of initial HBe Ag-positive patients in the ETV group relatively small compared to the TDF group (*P* = .086). So, the reduction rate of qHBs Ag in the ETV group was smaller than that in the TDF group, which may have been shown in the graph.

**Figure 3:**
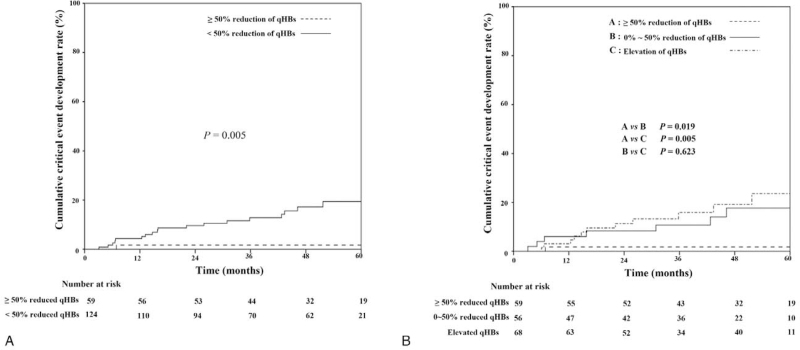
Cumulative critical event development in CHB patients according to the reduction of qHBs Ag Comparison of cumulative critical events between 50% reduction group and non-reduction group (A) and between 50% or more reduction group, 0∼50% reduction group, and elevation group (B).

Among the 22 patients with critical events, only 1 patient (4.5%) whose qHBs Ag level decreased by ≥50% after 1 year of antiviral therapy was included. Other patients with qHBs Ag ≥50% reduction, after 1 year of antiviral therapy had a rarer occurrence of critical events during the follow-up period compared to the group with a decrease of <50% or an increase (*P* = .003). Figure 3 presents the 5-year cumulative critical event development rate. The cumulative critical event development rate was significantly higher in the nonreduction group than in the reduction group (Fig. 3A). Alternatively, an analysis of the qHBs Ag levels divided by a ≥50% decrease (group A), 0 to 50% decrease (group B), and increased group (group C) revealed significant differences in the cumulative critical event development rate (Fig. 3B). The incidence of critical events for group A was significantly lower than that of groups B (*P* = .019) and C (*P* = .005). No significant difference was observed between groups B and C (*P* = .623).

### 3.3 Clinical characteristics of HCCs that developed during ETV or TDF therapy

In the 13 HCC patients, the median tumor size was 2.269 cm (range, 1.0–4.3 cm), and the number of tumors was one (100.0%) in HCC patients. All HCCs (100%) were of the nodular type, and the median AFP level at HCC diagnosis was 79.938 ng/mL. PVT was absent in all patients, and the BCLC stages were zero in 7 (53.8%), A in 5 (38.5%), and C in 1 (7.7%). Most (92.3%) were within the Milan criteria. Beyond the Milan criteria, HCC was observed in 1 patient. The patient was a 56-year-old man who had obstructive jaundice. The HCC was solitary, and the size was approximately 3.2 cm, but the tumor of this patient was an intrahepatic bile duct HCC (icteric-type HCC) containing confluence and right and left hepatic ducts.

### 3.4 Clinical characteristics of CHB patients with or without HCC

**Figure 4:**
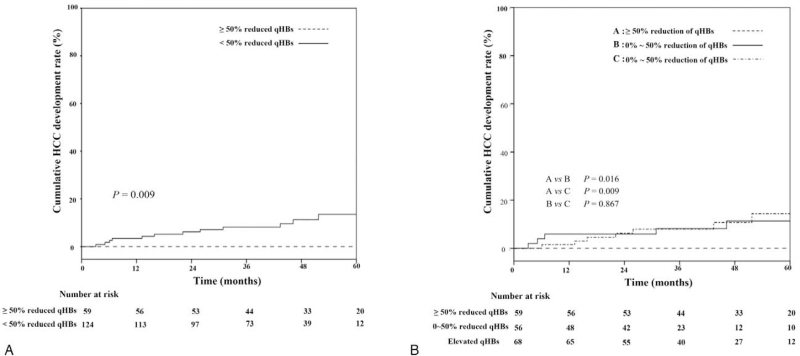
Cumulative hepatocellular carcinoma (HCC) development in CHB patients according to the reduction of qHBs Ag Comparison of cumulative HCC development between 50% reduction group and non-reduction group (A) and between 50% or more reduction group, 0∼50% reduction group, and elevation group (B).

Supplementary Table 1 presents the clinical baseline characteristics of the patients with or without HCC. Of the 183 study subjects, HCC developed in 13 patients after the start of antiviral therapy. None of the 13 patients with HCC showed a >50% reduction of qHBs Ag during the follow-up period (*P* = .010). Figure 4 shows the cumulative HCC development. The median age was similar (49.3 vs. 49.4). Among the patients with HCC, there were more males (76.9%), but the *P* value was not significant (*P* = .088). The quantified HBV DNA levels, the presence of HBe Ag, and its seroconversion after 1 year and cirrhosis did not significantly affect the occurrence of HCC (*P* values for all >0.05).

### 3.5 Predictors of HCC and critical event development in CHB patients treated with ETV or TDF

Univariate analysis of all 183 study subjects showed that the presence of LC at a base (hazard ratio [HR] 3.248, *P* < .015) and ≥50% reduction in the qHBs Ag level after 1 year of antiviral therapy (HR 0.085, *P* < .017) were associated with the occurrence of all critical events. Multivariate analysis showed that the presence of LC (HR 3.319, *P* = .016) and ≥50% reduction in the qHBs Ag level (HR 0.085, *P* = .018) independently predicted the occurrence of a critical event (Table 3).

**Table 3:** Significant predictive factors of critical events development during antiviral therapy.

	Univariate analysis		Multivariate analysis	
Variables	HR (95% CI)	*P*	HR (95% CI)	*P*
Age, y	0.987 (0.952–1.023)	.484		
Sex (male)	2.064 (0.799–5.332)	.134	1.833 (0.681–4.937)	.230
Albumin, mg/dL	0.632 (0.323–1.237)	.181		
Total bilirubin, mg/dL	0.925 (0.705–1.214)	.574		
PT, INR	3.620 (0.514–25.474)	.196		
LC, present	3.248 (1.255–8.407)	.015	3.319 (1.247–8.834)	.016
HBeAg, positive	0.745 (0.305–1.822)	.519		
HBeAg loss at 12 mo, yes, n (%)	0.563 (0.066–4.809)	.600		
HBV-DNA, copies/mL	1.000 (1.000–1.000)	.164		
AFP, ng/mL	0.997 (0.988–1.006)	.490		
AVT type (ETV vs TDF)	0.662 (0.251–1.743)	.404		
HBV-DNA negative at 12 mo, yes	1.160 (0.368–3.660) 0.800			
>50% Deceased HBsAg at 12 mo, yes, n (%)	0.085 (0.011–0.645)	0.017	0.085 (0.011–0.653)	.018

AFP = alpha-fetoprotein, ALT = alanine aminotransferase, CI = confidence interval, CTP = Child-Turcotte-Pugh classification, ETV = entecavir, HBs Ag = hepatitis B surface antigen, HR = hazard ratio, INR = international normalized ratio, LC = liver cirrhosis, PT = prothrombin time, TDF = tenofovir disoproxil fumarate.

A comparison of the cumulative HCC development and critical events between the ETV and TDF group revealed no significant difference (HCC, *p* = 0.179; critical events, *P* = .474) (Supplementary Figure 1). In addition, in the HBe Ag-positive and -negative groups, the qHBs Ag level showed a tendency to decrease less in the HBe Ag-negative group for 5 years, but it still tended to decrease continuously in both groups (Supplementary Figure 2).

## 4 Discussion

For decades, antiviral drugs with proven therapeutic effects in CHB patients have been developed and used to treat hepatitis B infections. On the other hand, even with treatment, CHB cannot be eradicated completely and the risk of HCC development still remained.^[25]^

Therefore, studies on the serological markers that can be used to monitor continuously the natural history, assess the treatment response, and predict the risk of liver-related complications in CHB patients are conducted alongside treatment. One method, HBs Ag quantification, is considered to be strongly related to the total amount of HBV DNA in the body, including HBV DNA in hepatocytes.^[26–29]^ In particular, qHBs Ag is known to be a predictor of the antiviral treatment response in patients receiving the PEG-IFN treatment.^[30–32]^ Although the qHBs Ag level could not predict the treatment response for patients treated with NAs unlike the PEG-IFN treatment,^[18,19,33,34,35]^ the qHBs Ag level is expected to have clinical implications related to the prognosis of CHB patients because it reflects the total amount transcribed from the integrated DNA and ccc DNA.^[36]^ When hepatitis B virus infects hepatocytes, it enters in the form of a relaxed circular DNA (rcDNA) genome or a double stranded linear DNA genome. The rcDNA is transformed into closed circular DNA (cccDNA), which serves as a template for replication and is inhibited by ETV or TDF when reverse transcribed in the hepatoplasm. The integrated DNA form is replication-defective, but it can drive carcinogenesis through processes such as genomic truncation and mutation. Viral proteins expressed from integrated HBV genomes have been shown to induce HCC spontaneously or by overexpression, and genomic truncation has shown that the function of HBx proteins is altered to induce carcinogenesis. In the early immune-tolerant phase, the number of infected hepatocytes increases through the replicative pathway using cccDNA. Hepatocytes with integrated DNA are relatively few. However, in the immune clearance phase, hepatocytes with cccDNA are reduced by the host immune system, and hepatocytes with integrated DNA survive and cause chronic infection by clonal expansion and new infection. This mechanism is the proposed process that HBs Ag titer may increase even after ETV or TDF treatment.^[32–34,37–38]^

But, among the present literature, few studies have compared and analyzed the correlation between qHBs Ag and HCC risk after highly potent antiviral drugs, such as ETV or TDF, in treatment-naive CHB patients. In addition, very few studies have individually analyzed HBe-Ag positive and -negative patients. This is notable in that the meaning of qHBs Ag was found to be different between HBe Ag positive and negative patients.^[20,35,39,40]^ The present study differs from the previous articles in that the predictive value of qHBs Ag was evaluated by including both HBe Ag-positive and -negative patients using ETV and TDF, which are presently used as primary treatment in the treatment of CHB. Treatment-naive HBe Ag-positive and -negative CHB patients were evaluated retrospectively through the changes in qHBs Ag. Patients showing a ≥50% decrease in the qHBs Ag levels after 1 year were relatively associated with fewer critical events and showed a lower incidence of HCC. Among the 183 study subjects, the qHBs Ag levels of 59 (32.2%) patients decreased by ≥50% after 1 year of antiviral treatment, and only 1 patient (1.7%) had critical events. Notably, patients with no cirrhosis and a rapid decrease in qHBs Ag within 1 year of therapy showed fewer critical events in the present study. In the 50% reduction group, no HCCs occurred during the 5-year follow-up period.

In the present study, the ALT value appeared to be a negative predictive value for the incidence of critical events (*P* = .019). However, this appears to be due to insurance standards for CHB treatment in Korea.^[10]^ As confirmed in the present study, patients with LC have a higher risk of critical events. According to the insurance policy for antiviral therapy initiation criteria in our country, CHB patients with cirrhosis are authorized to use ETV or TDF regardless of their ALT level. For CHB patients without cirrhosis, however, approval can be obtained only when the ALT level rises above the upper normal limit >2 or 3 times on the premise that there is no other cause for the rise. Therefore, in the present study, patients with CHB but without cirrhosis tended to have higher baseline ALT values before treatment. The follow-up period of patients in the non-50% reduction group was relatively short, presumably due to the higher incidence of HCC development or critical events in patients in this group.

The clinical significance of the qHBs Ag level revealed so far is the diagnosis of HBV infection, the definition of the disease phase, prediction of spontaneous HBs Ag seroconversion, prediction of reactivation in the inactive carrier state, and prediction of virological control after the discontinuation of NA treatment.^[41]^ In a meta-analysis conducted in China with HBe Ag-negative CHB patients treated with NAs, the probability of HBs Ag loss after stopping treatment was higher when the HBs Ag level decreased by ≥1 log IU/mL during 156 weeks of treatment (*P* < .001).^[42]^ In another systematic review, the virological and clinical relapse rates were estimated to be three times higher when the HBs Ag levels were more than 100 IU/mL at 12 to 24 months after the discontinuation of NA treatment.^[43]^ Despite the many studies on qHBs Ag, few studies have analyzed the risk of cumulative HCC and critical events according to the HBs Ag level after the start of ETV and TDF treatment in treatment-naive CHB patients. Therefore, the cutoff value of the HBs Ag level or clear guidelines for clinical applications has not been established. However, based on the previous studies in which the qHBs Ag level decreased from 0.16 to 0.3 log IU/mL per year during ETV and TDF therapy,^[44–47]^ the cutoff value was set to the value that conferred a 50% reduction after 1 year of treatment. Hence, a significant decrease in qHBs Ag in the first year of ETV and TDF treatment was associated with a low risk of critical events and HCC during the first 5 years of treatment.

The strength of our study is the pioneering analysis of qHBs Ag changes following ETV and TDF treatment in treatment-naive CHB patients, including both HBe Ag-positive and -negative patients. However, we also acknowledge that this study had several limitations. First, it was a single-center study, and the cohort size was relatively small. Although the number of CHB patients (n = 899) initially treated with ETV and TDF in our hospital was not small, the number of patients whose qHBs Ag level were regularly followed up at the initial and 6 months intervals was relatively insufficient. Second, since this study was only for Korean patients with genotype C HBV, there may be limitations in its application to patients with various HBV genotypes in the world. Third, there was a potential selective bias in our retrospective study. Therefore, we believe that a prospective, multinational multicenter study will be needed to support this study in the future.

In conclusion, the HBs Ag level decreased gradually during ETV and TDF therapy in both HBe Ag-positive and -negative CHB patients, and a significant decrease (≥50%) in the HBs Ag level during ETV and TDF treatment may indicate a lower risk of critical events, particularly HCC development. Therefore, monitoring the changes in HBs Ag level during ETV and TDF treatment may provide useful information for predicting the risk of complications in both HBe Ag-positive and -negative treatment-naive CHB patients.

## Author contributions

JH Lim, JH Yu, and YJ Jin were responsible for the concept and design of the study, the acquisition, analysis and interpretation of data, and drafting of the manuscript. JW Lee, and YJ Suh helped with data acquisition and interpretation of the data.

**Conceptualization:** Jung Hwan Yu, Jin-Woo Lee, Young-Joo Jin.

**Data curation:** Jung Hyun Lim, Jung Hwan Yu, Young Ju Suh, Jin-Woo Lee.

**Formal analysis:** Jung Hyun Lim, Young Ju Suh, Young-Joo Jin.

**Investigation:** Young-Joo Jin.

**Writing – original draft:** Jung Hyun Lim, Jung Hwan Yu.

## Supplementary Material

Supplemental Digital Content

## Supplementary Material

Supplemental Digital Content

## Supplementary Material

Supplemental Digital Content
