# Supplementary material for: Association between HBs Ag quantification and the risk of hepatocellular carcinoma in patients treated with tenofovir disoproxil fumarate or entecavir
Source: Medicine (Baltimore). 2021 Oct 1;100(39):e27417. doi: 10.1097/MD.0000000000027417 (PMC8483839; doi:10.1097/MD.0000000000027417)
Supplement: Supplemental Digital Content [file medi-100-e27417-s001.docx]

**Supplementary Table 1.** Characteristics of patients with and without HCC

| **Variables** | **Total**  **(n=183)** | **HCC group**  **(n=13)** | **No HCC group (n=170)** | ***P***^*^ |
| --- | --- | --- | --- | --- |
| Age (years) ^§^ | 49.4 (18-79) | 49.3 (26-70) | 49.4 (18-79) | 0.974 |
| Gender (male), n (%) | 97 (53.0) | 10 (76.9) | 87 (51.2) | 0.088 |
| ALT (IU/L) ^§^ | 162.0 (13-1871) | 63.4 (13-206) | 169.58 (14-1871) | 0.123 |
| Albumin (mg/dL) ^§^ | 4.02 (1.9-5.2) | 3.8 (2.3-4.6) | 4.03 (1.9-5.2) | 0.190 |
| T-bil (mg/dL) ^§^ | 1.47 (0.1-38.2) | 0.91 (0.3-2.0) | 1.51 (0.1-38.2) | 0.557 |
| PT, INR ^§^ | 1.12 (0.87-2.07) | 1.15 (0.9-1.6) | 1.12 (0.87-2.07) | 0.514 |
| AFP (ng/mL) ^§^ | 42.9 (0.8-1,391) | 24.3 (1.4-202) | 44.4 (0.8-1,391) | 0.674 |
| LC, present, n (%) | 79 (43.2) | 8 (61.5) | 71 (41.8) | 0.245 |
| CTP class, A/B/C, n (%) | 154/27/2  (84.2/14.8/1.0) | 9/4/0  (69.2/30.8/0) | 145/23/2  (85.3/13.5/1.2) | 0.220 |
| HBeAg, positive, n (%) | 95 (51.9) | 6 (46.2) | 89 (52.4) | 0.777 |
| HBeAg loss at 12 months, yes, n (%) | 15 (8.2) | 1 (7.7) | 14 (8.2) | 1.000 |
| HBV-DNA (copies/mL) ^§^ | 3.16x10⁸  (57-3.41x10⁹) | 2.44x10⁷  (145-1.50x10⁸) | 3.38x10⁸  (57-3.41x10⁹) | 0.173 |
| HBV-DNA negative  at 12mo, yes, n (%) | 146 (79.8) | 11 (84.6) | 135 (79.4) | 1.000 |
| >50% deceased HBs Ag at 12mo, yes, n (%) | 59 (32.2) | 0 | 59 (34.7) | 0.010 |

^§^, median (range)

^*,^ *P* values were calculated using the *t*-test or *chi*-square test between the HCC and no HCC group

^+^, Fisher’s exact test
