# Supplementary material for: Association between HBs Ag quantification and the risk of hepatocellular carcinoma in patients treated with tenofovir disoproxil fumarate or entecavir
Source: Medicine (Baltimore). 2021 Oct 1;100(39):e27417. doi: 10.1097/MD.0000000000027417 (PMC8483839; doi:10.1097/MD.0000000000027417)
Supplement: Supplemental Digital Content [file medi-100-e27417-s002.doc]

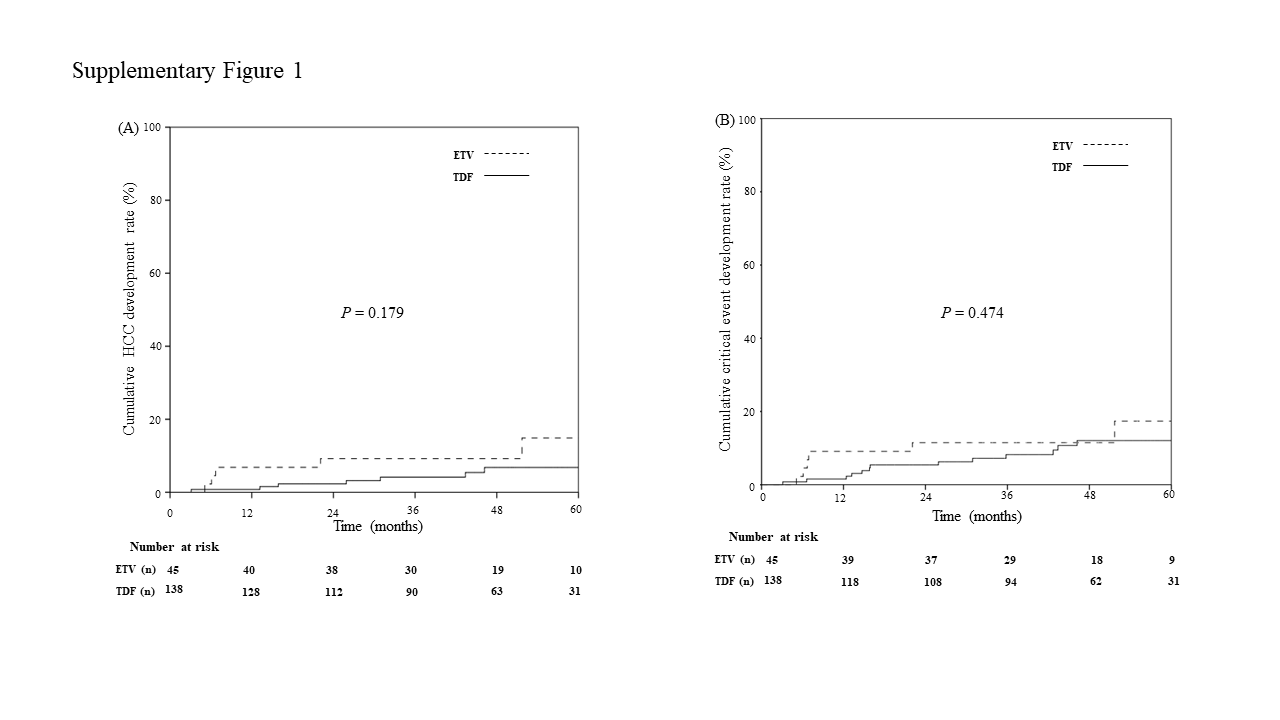


**Supplementary Figure 1. Cumulative HCC and critical event development in ETV and TDF group** Comparison of cumulative HCC development (a) and cumulative critical event development (b) between ETV and TDF patients groups.
