# Supplementary material for: Association between HBs Ag quantification and the risk of hepatocellular carcinoma in patients treated with tenofovir disoproxil fumarate or entecavir
Source: Medicine (Baltimore). 2021 Oct 1;100(39):e27417. doi: 10.1097/MD.0000000000027417 (PMC8483839; doi:10.1097/MD.0000000000027417)
Supplement: Supplemental Digital Content [file medi-100-e27417-s003.doc]

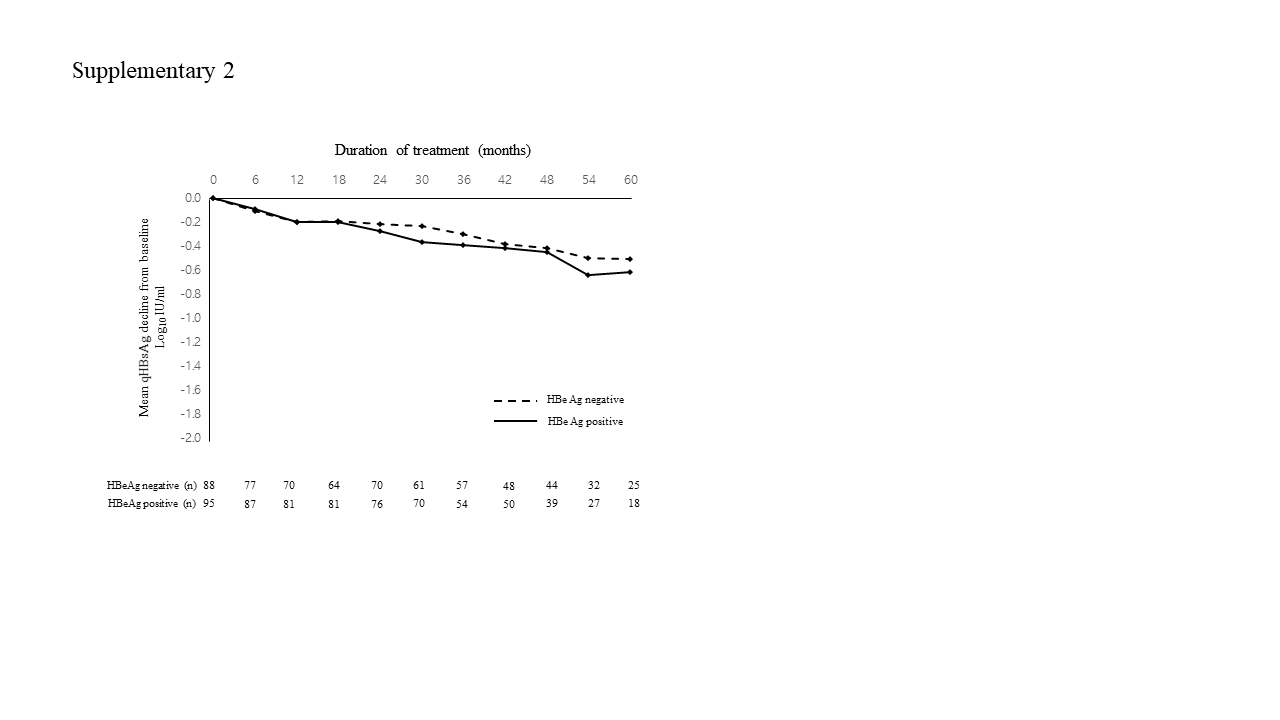


**Supplementary Figure 2. Mean qHBs Ag decline from baseline in hepatitis B envelope antigen (HBe Ag) positive and negative patients** Comparison of mean qHBs Ag decline from baseline between HBe Ag positive and negative patients groups.
